# Targeted Blood Brain Barrier Opening With Focused Ultrasound Induces Focal Macrophage/Microglial Activation in Experimental Autoimmune Encephalomyelitis

**DOI:** 10.3389/fnins.2021.665722

**Published:** 2021-05-12

**Authors:** Katharina Schregel, Caroline Baufeld, Miklos Palotai, Roberta Meroni, Paolo Fiorina, Jens Wuerfel, Ralph Sinkus, Yong-Zhi Zhang, Nathan McDannold, P. Jason White, Charles R. G. Guttmann

**Affiliations:** ^1^Department of Radiology, Brigham and Women’s Hospital, Harvard Medical School, Boston, MA, United States; ^2^Department of Neuroradiology, Heidelberg University Hospital, Heidelberg, Germany; ^3^Institute of Neuroradiology, University Medical Center Göttingen, Göttingen, Germany; ^4^Ann Romney Center for Neurologic Diseases, Department of Neurology, Brigham and Women’s Hospital, Harvard Medical School, Boston, MA, United States; ^5^Nephrology Division, Boston Children’s Hospital, Harvard Medical School, Boston, MA, United States; ^6^Department of Anesthesia and Intensive Care, IRCCS San Raffaele Scientific Institute, Milan, Italy; ^7^Transplantation Research Center, Renal Division, Brigham and Women’s Hospital, Boston, MA, United States; ^8^International Center for T1D, Pediatric Clinical Research Center Fondazione Romeo ed Enrica Invernizzi, Department of Biomedical and Clinical Science L. Sacco, University of Milan, Milan, Italy; ^9^MIAC AG and Department of Biomedical Engineering, University Basel, Basel, Switzerland; ^10^Division of Imaging Sciences & Biomedical Engineering, King’s College London, London, United Kingdom; ^11^INSERM UMR S1148 - Laboratory for Vascular Translational Science, University Paris, Paris, France

**Keywords:** experimental autoimmune encephalomyelitis, focused ultrasound, blood brain barrier, magnetic resonance imaging, microglia, magnetic resonance elastography

## Abstract

Experimental autoimmune encephalomyelitis (EAE) is a model of multiple sclerosis (MS). EAE reflects important histopathological hallmarks, dissemination, and diversity of the disease, but has only moderate reproducibility of clinical and histopathological features. Focal lesions are less frequently observed in EAE than in MS, and can neither be constrained to specific locations nor timed to occur at a pre-specified moment. This renders difficult any experimental assessment of the pathogenesis of lesion evolution, including its inflammatory, degenerative (demyelination and axonal degeneration), and reparatory (remyelination, axonal sprouting, gliosis) component processes. We sought to develop a controlled model of inflammatory, focal brain lesions in EAE using focused ultrasound (FUS). We hypothesized that FUS induced focal blood brain barrier disruption (BBBD) will increase the likelihood of transmigration of effector cells and subsequent lesion occurrence at the sonicated location. Lesion development was monitored with conventional magnetic resonance imaging (MRI) as well as with magnetic resonance elastography (MRE) and further analyzed by histopathological means. EAE was induced in 12 6–8 weeks old female C57BL/6 mice using myelin oligodendrocyte glycoprotein (MOG) peptide. FUS-induced BBBD was performed 6, 7, and 9 days after immunization in subgroups of four animals and in an additional control group. MRI and MRE were performed on a 7T horizontal bore small animal MRI scanner. Imaging was conducted longitudinally 2 and 3 weeks after disease induction and 1 week after sonication in control animals, respectively. The scan protocol comprised contrast-enhanced T1-weighted and T2-weighted sequences as well as MRE with a vibration frequency of 1 kHz. Animals were sacrificed for histopathology after the last imaging time point. The overall clinical course of EAE was mild. A total of seven EAE animals presented with focal T2w hyperintense signal alterations in the sonicated hemisphere. These were most frequent in the group of animals sonicated 9 days after immunization. Histopathology revealed foci of activated microglia/macrophages in the sonicated right hemisphere of seven EAE animals. Larger cellular infiltrates or apparent demyelination were not seen. Control animals showed no abnormalities on MRI and did not have clusters of activated microglia/macrophages at the sites targeted with FUS. None of the animals had hemorrhages or gross tissue damage as potential side effects of FUS. EAE-animals tended to have lower values of viscoelasticity and elasticity in the sonicated compared to the contralateral parenchyma. This trend was significant when comparing the right sonicated to the left normal hemisphere and specifically the right sonicated compared to the left normal cortex in animals that underwent FUS-BBBD 9 days after immunization (right vs. left hemisphere: mean viscoelasticity 6.1 vs. 7.2 kPa; *p* = 0.003 and mean elasticity 4.9 vs. 5.7 kPa, *p* = 0.024; right vs. left cortex: mean viscoelasticity 5.8 vs. 7.5 kPa; *p* = 0.004 and mean elasticity 5 vs. 6.5 kPa; *p* = 0.008). A direct comparison of the biomechanical properties of focal T2w hyperintensities with normal appearing brain tissue did not yield significant results. Control animals showed no differences in viscoelasticity between sonicated and contralateral brain parenchyma. We here provide first evidence for a controlled lesion induction model in EAE using FUS-induced BBBD. The observed lesions in EAE are consistent with foci of activated microglia that may be interpreted as targeted initial inflammatory activity and which have been described as pre-active lesions in MS. Such foci can be identified and monitored with MRI. Moreover, the increased inflammatory activity in the sonicated brain parenchyma seems to have an effect on overall tissue matrix structure as reflected by changes of biomechanical parameters.

## Introduction

Experimental autoimmune encephalomyelitis (EAE) is a widely used model of multiple sclerosis (MS) and shares important histopathological features with this disease: inflammation, demyelination, axonal loss and gliosis (Constantinescu et al., 2011). The model has been used to develop and test drugs now approved for use in humans (Denic et al., 2011). EAE can be induced in several species applying either active immunization with CNS tissue or myelin peptides or adoptive transfer of encephalitogenic T-cells (Kipp et al., 2012). In EAE, myelin-specific T-cells cross the blood brain barrier and induce a cascade of inflammatory processes, which, amongst other effects, lead to blood brain barrier disruption (BBBD; Muller et al., 2005). Consequently, more activated immune cells penetrate into the brain and further enhance neuroinflammation, ultimately resulting in demyelination, and axonal damage (Fletcher et al., 2010). EAE lesions develop predominantly in the spinal cord, but also in the brain stem, hindbrain and cortex, and diffuse perivascular inflammation, meningitis, or optic neuritis can also be observed (Gold et al., 2006). As much as EAE reflects the dissemination and diversity of MS pathology, focused analysis of lesion development and progression or correlation of structural and functional deficits is hindered (Kerschensteiner et al., 2004). Hence, several groups have worked on the development of a localized model, in which lesions can be targeted to specific brain or spinal cord regions of interest. The induction of focal inflammatory lesions has been accomplished by focal thermal injury (Levine and Hoenig, 1968), intracerebral injections of gliotoxic agents (Woodruff and Franklin, 1999) or heat-killed mycobacteria (Newman et al., 2001) or by injection of activated T-cells (Westland et al., 1999) or lipopolysaccharide (Felts et al., 2005) into the spinal cord. However, the pathomechanism and appearance of lesions induced with such methods clearly differ from MS lesions. Therefore, further research aimed to establish a model of focal lesion development that resembles MS pathology more closely is needed. Kerschensteiner et al. (2004) created a targeted EAE model by injecting a cytokine solution in the spinal cord of rats sensitized with recombinant myelin oligodendrocyte glycoprotein (MOG; Ineichen et al., 2017). Even though lesions reflected key features of MS pathology and could be reliably targeted to specific white matter tracts in the spinal cord (Kerschensteiner et al., 2004), this model is invasive and requires spinal cord surgery.

A transient BBBD can also be induced by combining focused ultrasound (FUS) with an intravenous injection of encapsulated gas-filled microbubbles that are commercially available as an ultrasound contrast agent (Vykhodtseva et al., 2008; Jolesz and McDannold, 2014). The BBBD is only evoked in the focal volume of the ultrasound transducer, possibly caused by radiation forces stemming from the oscillatory and/or acoustic streaming effects of the microbubbles (Bing et al., 2009).

Neuroinflammation, demyelination as well as axonal and neuronal damage interfere with the normal geometrical network of brain parenchyma. This is quantifiable using magnetic resonance elastography (MRE) and changes of cerebral biomechanics have been demonstrated in animal models (Riek et al., 2012; Schregel et al., 2012; Millward et al., 2015; Wang et al., 2020) and in patients with MS (Wuerfel et al., 2010; Streitberger et al., 2012; Fehlner et al., 2015). Briefly, MRE is a phase contrast-based magnetic resonance imaging (MRI) technique that measures tissue displacement caused by propagating mechanical shear waves (Muthupillai and Ehman, 1996; Manduca et al., 2020). Biomechanical properties can be inferred from the displacement field (Fovargue et al., 2018) and allow for the quantification of tissue stiffness, for example. Thus, MRE renders information on the microstructure of the brain parenchyma that is not detectable with conventional MRI.

In this study, we transiently disrupted the BBB in mice immunized with MOG to develop a controlled model of focal brain lesions in EAE. We hypothesized that FUS will increase the likelihood of lesion occurrence in those areas where the BBB has been opened, as this may facilitate the influx of inflammatory immune cells into the brain. Lesion development was monitored with conventional MRI and MRE and further analyzed by histopathological means. This work is intended as a proof-of-concept study to describe a method for focal lesion induction in EAE and to present first results.

## Materials and Methods

### Animals

All experiments were performed in accordance with the local institutional animal care and use committee (IACUC). A total of 16 healthy female 6–8 weeks old C57BL/6 mice (Jackson Laboratories, Bar Harbor, ME, United States) were housed in a climate-controlled room with a 12h/12h light-dark cycle and food and water *ad libitum*. EAE was induced in 12 animals using MOG peptide 35–55 as previously described (Bittner et al., 2014). MOG35–55 was used at a concentration of 1.5 mg/ml dissolved in phosphate-buffered saline (PBS). The solution was emulsified in an equal volume of Complete Freund’s Adjuvant (CFA). Mice were injected with 100 μl s.c. in each flank (150 μg MOG35–55 in total per mouse). Additionally, 200 μl pertussis toxin (PTX LOT# 180235A1A) dissolved in PBS at a concentration of 1 ng/μl was injected i.p. at immunization and 48 h later (200 ng pertussis toxin per mouse per injection). The remaining four mice were not immunized and served as controls. All mice were examined, weighed and scored for clinical symptoms daily as follows: grade 0—no abnormality; grade 1—limp tail or impaired righting attempt, grade 2—partial hind limb paralysis (paraparesis); grade 3—total hind limb or partial hind and front limb paralysis (paraplegia); grade 4—total hind and partial front limb paralysis (quadriplegia); grade 5—moribund or dead animals (Beeton et al., 2007; Smorodchenko et al., 2007). Mice that were in between the graduations of clinical symptoms were given intermediate scores in increments of 0.5. Upon clinical occurrence of severe symptoms (grade 3), animals were provided with food within the cage and gel packs to ensure access to fluids.

### Focused Ultrasound

**FIGURE 1:**
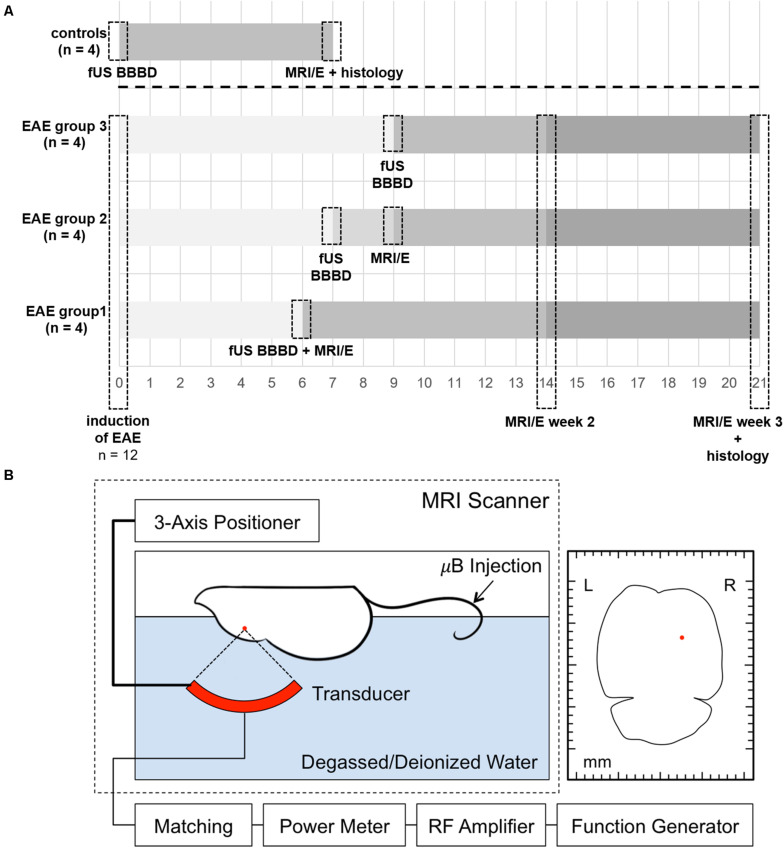
Timeline of the experiments and schematic overview of the FUS set-up. A timeline summarizing the experimental procedures for EAE animals and controls is shown in panel **(A)**. EAE was induced by immunization with MOG in 12 healthy female C57BL/6 mice. FUS was performed 6, 7, or 9 days after EAE induction, respectively, in subgroups of four animals each. A subgroup of four animals (EAE group 1) was scanned immediately after sonication on day 6 after immunization to confirm BBBD. Another subgroup (EAE group 2) was imaged 2 days after sonication (9 days after immunization) to study potential early effects. All EAE animals received longitudinal MRI and MRE (MRI/E) scans 2 and 3 weeks after immunization. Control animals received only one MRI and MRE scan 7 days after FUS BBBD. Histological analyses were performed after the last imaging time point. For FUS **(B)**, the animal is placed in supine position on a custom-built FUS device with its head fixated in a stereotactic frame. A three-axis positioner allows accurate planning of the area that should be sonicated. The red dot in the axial schematic of the mouse brain on the right indicates the targeted area. Acoustic coupling was achieved by submersing the top of the animal’s head and a 690 kHz FUS transducer in degassed and deionized water. The spherically curved transducer was driven with a function generator and an amplifier. Sonication was started immediately after the i.v. administration of a microbubble ultrasound contrast agent in the tail vein (Optison, GE Healthcare, Little Chalfont, Buckinghamshire, United Kingdom; dose 100 μl/kg, diluted 10× in PBS). μB, microbubbles.

Animals were anesthetized with an i.p. injection of ketamine (100 mg/kg) and xylazine (10 mg/kg). The head was shaved and depilated, and a catheter was placed in the tail vein. Then, the animal was placed in supine position on a custom-built FUS device with its head fixated in a stereotactic frame. Acoustic coupling was achieved by submersing the top of the animal’s head and a 690 kHz FUS transducer in degassed and deionized water. The spherically curved transducer was driven with a function generator and an amplifier. The transducer was attached to a manual fine positioning system and FUS was planned to cover the lateral corpus callosum as well as the adjacent cortex and basal ganglia of the right hemisphere. Peak negative pressures between 260 and 270 kPa were delivered in 10-ms pulses (6900 x λ) at a pulse-repetition frequency of 2 Hz for 95 s. Sonication was started immediately after the i.v. administration of a microbubble ultrasound contrast agent (Optison, GE Healthcare, Little Chalfont, Buckinghamshire, United Kingdom; dose 100 μl/kg, diluted 10× in PBS). FUS was performed 6, 7, or 9 days after EAE induction, respectively, in subgroups of four animals each (see Figure 1A for a timeline of the experiment and Figure 1B for a schematic of the FUS set-up). In order to maximize the likelihood of effector cells transmigrating into brain parenchyma upon FUS-BBBD, we targeted the period of peaking cellular response in the blood, as reported in the literature (Barthelmes et al., 2016). While first symptoms of EAE typically occur 9 – 14 days after immunization with MOG (Bittner et al., 2014), a transient increase of immune cells in the blood was described to take place in the preclinical phase of MOG-induced EAE (Barthelmes et al., 2015, 2016). Furthermore, altered BBB permeability and tight junction pathology in spinal cord vessels have been observed prior to symptom occurrence 7 days after EAE-induction with MOG (Bennett et al., 2010). Based on this, we scheduled FUS in a period were effector cells are increased in the blood and immune activation is ongoing.

### MRI and MRE

Magnetic resonance imaging was performed on a 7T horizontal bore Bruker small animal scanner (Ettlingen, Germany; gradient strength 660 mT/m) using an 8.6 cm body coil for transmission and a 2-cm surface coil for reception. MRI and MRE data were acquired longitudinally 2 and 3 weeks after induction in all EAE-animals (EAE groups 1, 2, and 3). A subgroup of four animals was scanned immediately after sonication on day 6 after immunization to confirm BBBD (EAE group 1). Another subgroup was imaged 2 days after sonication (9 days after immunization) to study potential early effects (EAE group 2). Control animals received only one MRI scan 7 days after FUS (Figure 1A). Before imaging, 100 μl gadopentetate dimeglumine contrast agent (Magnevist, Bayer Health Care LLC, Whippany, NJ, United States) were administered intraperitoneally. Anesthesia was then induced with 2.5% isoflurane in 100% O_2_ and maintained with 1–1.5% isoflurane in 100% O_2_ delivered via a nose cone during the imaging procedure. Respiration rate was constantly monitored (SA Instruments, Stony Brook, NY, United States) and body temperature was sustained using a heated water mattress. Animals were placed prone on a custom-build MRE bed (Schregel et al., 2018, 2020). Briefly, the bed includes a cage-assembly for head fixation, which is connected to an external transducer outside the MRI scanner via a flexible rod. The rod transmits mechanical vibrations generated by the transducer and causes a rocking motion of the head cage, ultimately leading to the transmission of mechanical shear waves to the animal’s brain. After standard calibration, pilot scans and shimming, the following sequences were acquired: A coronal T1-weighted (T1w) sequence (FLASH; TR/TE 250/5.4 ms; FOV 19.2; matrix 192 × 192; six averages; nine 0.3 mm-thick slices; acquisition time 3 min 36 s), an axial T1w sequence (RARE; TR/TE 1300/9 ms; FOV 20; matrix 128 × 128; six averages; 20 0.3 mm thick slices; acquisition time 6 min 14 s), a coronal T2-weighted (T2w) sequence (RARE; TR/TE 5000/56 ms; FOV 19.2; matrix 192 × 192; six averages; nine 0.3 mm-thick slices; acquisition time 12 min), and a coronal T2^∗^-weighted (T2^∗^w) sequence (GEFC; TR/TE 395/20 ms; FOV 19.2; matrix 192 × 192; six averages; nine 0.3 mm-thick slices; acquisition time 7 min 35 s). Conventional MRI was followed by a customized multi-slice, single spin echo MRE sequence (TR/TE 900/29 ms; FOV 19.2 mm; matrix 64 × 64; one average; eight wave phases; nine slices; isotropic resolution 0.3 mm; vibration frequency 1 kHz; acquisition time 23 min). T1w images were acquired 30–40 min after i.p. injection of contrast agent. The coronal sequences were positioned in the sonicated region and covered identical volumes. The axial T1w sequence covered the entire brain.

T1w, T2w, and T2^∗^w images were analyzed using open source 3D Slicer software (version 4.6, www.slicer.org) (Fedorov et al., 2012). Number and location of conspicuities on T2w scans were noted independently by two raters, blinded to histopathological findings. The raters agreed in almost all cases. In the remaining cases, the two raters reached consensus by reviewing discrepancies together over video conference. Consensus results are reported. Contrast-enhancement was evaluated qualitatively using both axial and coronal T1w images. Additionally, T2^∗^w scans were screened for presence of micro- or macrohemorrhages. MRE data were reconstructed and evaluated with dedicated in-house software (ROOT environment, CERN, Meyrin, Switzerland). T2w images were displayed in the same software and used as anatomical reference. Regions-of-interest (ROIs) covering the right sonicated as well as the left hemispheres, cortices, corpus callosum, and deep gray matter were defined on T2w images and copied to the reconstructed MRE maps. Additional ROIs covered focal signal abnormalities on T2w images and a corresponding area in the contralateral normal appearing brain tissue (NABT). Mean and standard deviation were calculated for the following MRE parameters: the absolute value of the complex valued shear modulus | G^∗^|, which comprises measures of elasticity *G*_*d*_ and viscosity *G*_*l*_ and is termed viscoelasticity in the following ($\left|G^{{}^{*}}\right|=\sqrt{G_{d}^{2}+G_{l}^{2}}$), elasticity *G*_*d*_, viscosity *G*_*l*_ and the shear modulus phase angle Y ($Y=\frac{2}{\pi }\,\text{atan}\,\left(\frac{G_{l}}{G_{d}}\right)$), which reveals whether a tissue behaves more like an elastic (Y = 0) or a viscous material (Y = 1).

### Histology

Mice were deeply anesthetized, euthanized and tissue was fixed via transcardial perfusion of 10 ml saline followed by 10% neutral buffered paraformaldehyde (PFA; Sigma-Aldrich, St Louis, MO, United States). Brains were harvested, post-fixed in 10% neutral buffered PFA and prepared for paraffin sectioning. Paraffin-embedded brains were cut in 5-μm coronal sections so that the sections covered the same area as the MR images. Sections were stained by standard H&E and LFB staining to assess inflammation and demyelination, respectively. Additionally, immune-histochemical staining for activated microglia/macrophages was accomplished after deparaffinization and heat induced antigen retrieval using anti-Iba1-antibody (dilution 1:500; 1 μg/ml; WAKO Chemicals, Richmond, VA, United States). In addition to Iba-1 immunohistochemistry, we performed immunohistochemistry for TMEM119, which is highly specific for resident microglia and thus gives us the possibility to distinguish microglia from peripheral cells (anti-TMEM119-antibody, dilution 1:100; 0.153 mg/ml; Abcam, Cambridge, MA, United States) (Butovsky et al., 2014; Bennett et al., 2016; Satoh et al., 2016). Sections were examined with light microscopy and digitally photographed and processed using BIOQUANT LifeScience 2012 software (BIOQUANT Image Analysis Corporation, Nashville, TN, United States). An independent investigator blinded to the results from MR imaging performed the histological analyses and examined the stained slices for presence of cellular infiltration, demyelination, tissue damage, hemorrhage and activated microglia/macrophages. The analysis focused on sections that were covered in the MRI exams and hence included the sonicated area.

### Statistical Analysis

The data were analyzed using GraphPad Prism 9 (GraphPad Software, La Jolla, CA, United States). MRI and histopathological data were analyzed descriptively. Numbers and percentages are reported. Two-way analyses of variance (ANOVA) followed by Bonferroni’s test for multiple comparisons were conducted on the influence of sonication and group on the cerebral MRE-parameters. Sonication differentiated the right (sonicated) from the left (normal) hemisphere. Group included the three subsets of EAE-animals sonicated at different time points and the control animals. In addition, a separate two-way ANOVA followed by Bonferroni’s test for multiple comparisons was performed in order to evaluate longitudinal changes of the biomechanical properties. For this, data acquired in the subgroup of animals sonicated 6 and 9 days after EAE-induction were analyzed. The differences in MRE-parameters of T2w-conspicuities and NABT were evaluated using a paired *t*-test. The significance level of all tests was set to α = 0.05. Adjusted *p*-values derived from Bonferroni’s test and *p*-values from the paired *t*-test are reported. The data that support the findings of this study are available from the corresponding author, upon reasonable request.

## Results

### The Overall Clinical Course of EAE Was Mild

The clinical course of EAE was mild in most animals and the first clinical symptoms occurred 11–20 days (mean 14.6, 95% CI 9.74–19.46) after immunization. Impaired motility of the tail was most commonly observed and progressed to a maximum severity of complete paraplegia of the hind limbs. Control animals did not develop any clinical symptoms. One animal died after the first MRI due to complications of anesthesia and another one had to be sacrificed prematurely because of a moribund clinical condition.

### T2-Weighted Signal Alterations Were Observable in the Sonicated Hemisphere of Animals With EAE

**FIGURE 2:**
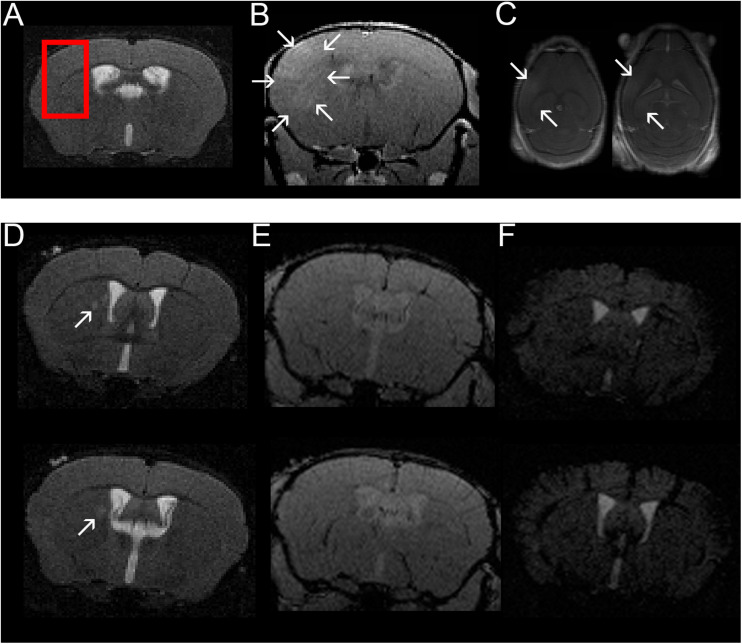
Confirmation of BBBD in the area targeted with FUS using contrast-enhanced MRI and example of one mouse with a T2w hyperintense lesion in the right thalamus. A coronal T2w- **(A)** and coronal **(B)** and axial **(C)** contrast-enhanced T1w images are shown (images from animal 1 in Tables 1, 2). FUS-BBBD was targeted to cover the lateral corpus callosum as well as the adjacent cortex and deep gray matter of the right hemisphere [red box in panel **(A)**]. Successful BBBD was confirmed using contrast-enhanced T1w sequences. The arrows in panels **(B,C)** indicate diffusely contrast-enhancing parenchyma within the sonicated region. Of note, the ventricular hyperintensity in panels **(B,C)** is likely caused by leakage of contrast agent into the CSF due to disruption of the blood-CSF-barrier. Two consecutive T2w **(D)**, contrast-enhanced T1w **(E)** and T2*w **(F)** sections of a mouse sonicated 9 days after EAE induction with MOG are shown (animal 11 in Tables 1, 2). The MRI scan was acquired 14 days after immunization (5 days after FUS-BBBD). On the T2w images, an oval shaped hyperintense conspicuity with narrow hypointense rim is visible in the right thalamus [arrows in panel **(D)**]. The T1w images **(E)** do not show any distinct contrast enhancement at the corresponding location. Of note, the ventricular hyperintensity in panel **(E)** is likely caused by leakage of contrast agent into the CSF due to disruption of the blood–CSF-barrier. There are no hypointense signal alterations indicative of intraparenchymal bleedings visible on the T2*w images **(F)**. The tubular hypointense structures visible in the cortex for example represent blood vessels.

A subgroup of four animals received a contrast-enhanced MRI immediately after FUS. All animals showed contrast-enhancement in the cortex, lateral corpus callosum and deep gray matter of the right hemisphere indicating a successful BBBD in these sonicated locations (Figures 2A–C). All MRI scans acquired were examined and screened for signal alterations and contrast enhancement (Table 1). A total of seven EAE animals (7/12, 58.3 %) presented with conspicuities on T2-weighted (T2w) MRI that were performed 2 and 3 weeks after immunization, respectively. All conspicuities were located in the right hemisphere within the sonicated area. Two animals presented with two findings, while the others had one finding each. The conspicuities had a similar appearance and configuration in most animals (6/7, 85.7%): they had a round or oval shape, a diameter of a few millimeters, were hyperintense on T2w and had a narrow T2w-hypointense rim (Figure 2D). In one animal (1/7, 14.3%), the finding had a T2w-hypointense center with a hyperintense rim on the first scan (2 weeks post-immunization), but appeared hyperintense with hypointense rim on the second scan (3 weeks post-immunization). In three animals (3/7, 42.9%), the conspicuities were visible on both MRI exams. One animal (1/7, 14.3%) had a signal alteration on the T2w images acquired 2 days after sonication. The finding could not be followed longitudinally, as this mouse died after the MRI scan due to complications of the anesthesia. The remaining three animals (3/7, 42.9%) presented with findings on T2w scans after 3 weeks. When comparing the three FUS groups, the T2w signal alterations were most frequent in the group that was sonicated 9 days after EAE induction (4/4, 100%). Half of the animals (2/4, 50%) that were sonicated 7 days after immunization had MRI findings, while only one animal (1/4, 25%) showed T2w abnormalities when FUS was performed 6 days after EAE induction. The findings were mostly T1w-isointense to the surrounding brain parenchyma and did not show any clear contrast enhancement (Figure 2E). The conspicuities were predominantly observed in the gray matter. The most common locations were the right striatum (4/9, 44.4%) and the right cortex (4/9, 44.4%) followed by the right thalamus (1/9, 11.2%). Distinct callosal lesions could not be seen. The brain of control animals appeared normal on all MRI acquired and showed neither conspicuities on T2w MRI nor pathological contrast-enhancement of the brain tissue on T1w MRI. The T2^∗^w images of both EAE and control animals were screened for hemorrhages. No animal (0/16, 0%) presented with abnormal T2^∗^w-hypointensities (Table 1 and Figure 2F) and hence bleedings as potential side effect of FUS could be excluded.

**TABLE 1:** Summary of MRI findings in animals with EAE and in healthy controls.

Animal	FUS performed after immunization (days)	Number of T2w-hyperintense lesions			Contrast-enhancement			T2*w signal alteration		
		d9 MRI	d14 MRI	d21 MRI	d9 MRI	d14 MRI	d21 MRI	d9 MRI	d14 MRI	d21 MRI
**EAE**										
1*	6	n/a	0	1	n/a	0	0	n/a	0	0
2	6	n/a	0	0	n/a	0	0	n/a	0	0
3	6	n/a	0	0	n/a	0	0	n/a	0	0
4	6	n/a	n/a	n/a	n/a	0	0	n/a	0	0
5	7	0	0	0	0	0	0	0	0	0
6*	7	0	1	2	0	0	0	0	0	0
7	7	1	n/a	n/a	0	0	0	0	0	0
8	7	0	0	0	0	0	0	0	0	0
9*	9	n/a	0	2	n/a	0	0	n/a	0	0
10	9	n/a	0	1	n/a	0	0	n/a	0	0
11*^#^	9	n/a	1	1	n/a	0	0	n/a	0	0
12	9	n/a	1	1	n/a	0	0	n/a	0	0
**Control**										
1	n/a	n/a	0	n/a	n/a	0	n/a	n/a	0	n/a
2	n/a	n/a	0	n/a	n/a	0	n/a	n/a	0	n/a
3	n/a	n/a	0	n/a	n/a	0	n/a	n/a	0	n/a
4	n/a	n/a	0	n/a	n/a	0	n/a	n/a	0	n/a

*The asterisks indicate the animals with hyperintense T2w-lesions that could be co-localized to clusters of activated macrophages and microglia. The hashtag indicates the animal with a T2w-lesion that could be co-localized to a cluster of activated Iba1- and TMEM119-positive resident microglia. FUS, focused ultrasound.*

### Biomechanical Properties Changed Within the Sonicated Hemisphere and Cortex of Animals With EAE

**FIGURE 3:**
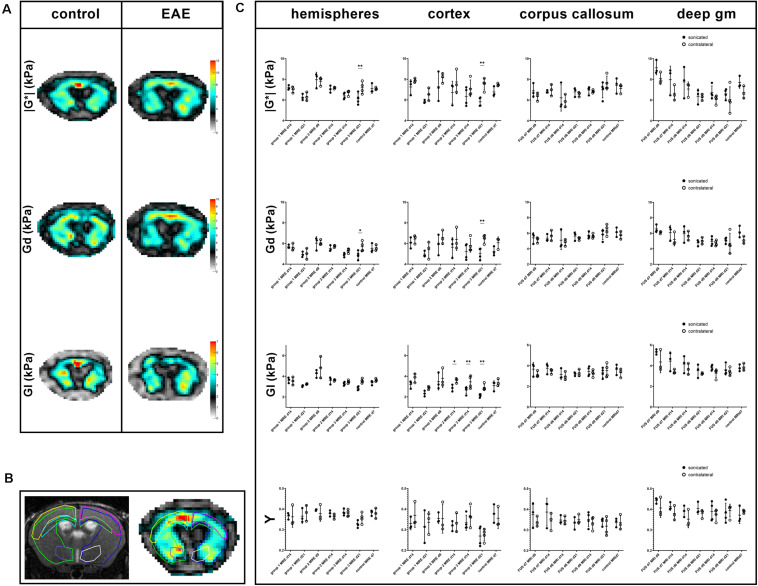
Exemplary MRE-maps of a control mouse and an animal with EAE, regions of interest and comparison of MRE-parameters. Maps of viscoelasticity lG*l, elasticity *G*_*d*_, viscosity *G*_*l*_ of a healthy control animal [(**A,** left panel); control animal 3 in Tables 1, 2] are rather symmetric. In contrast, the MRE-maps of an animal of EAE-group 3 that was sonicated 9 days after immunization show some asymmetry [(**(A,** right panel); animal 12 in Tables 1, 2]. Viscoelasticity, elasticity, and viscosity of the right sonicated hemisphere appear to be lower than on the contralateral side. All MRE-parameters were quantitatively assessed in regions-of-interest (ROIs). These were defined on T2w images and copied to the MRE-maps. **B** shows a T2w image on the left and the corresponding lG*l-map of a control animal on the right with ROIs in different colors: the right sonicated as well as the left hemispheres (light green and blue), cortices (yellow and pink), corpus callosum (light blue and green) and deep gray matter (purple and white). Mean values of the MRE-parameters | G*|, *G*_*d*_, *G*_*l*_ and phase angle Y were compared between the sonicated [**(C)**, black dots] and the contralateral [**(C)**, white dots] regions. As already visible in the MRE-maps, in EAE group 3-animals values of viscoelasticity | G*| and elasticity *G*_*d*_ were lower in the sonicated right hemisphere and cortex compared to the contralateral side 21 days after immunization. Moreover, viscosity *G*_*l*_ of the right sonicated cortex was lower in animals in EAE group 3 both 14 and 21 days after immunization. Similarly, *G*_*l*_ of the sonicated cortex was lower in group 2 EAE-animals. The shear modulus phase angle Y did not show different values in the right sonicated compared to the left normal structures. FUS-BBBD itself did not seem to affect cerebral biomechanics, as the MRE-parameters of control animals were similar in the sonicated and the contralateral brain parenchyma. The asterisks in panel **(C)** indicate level of significance derived from Bonferroni’s test for multiple comparisons, which followed a two-way ANOVA. deep gm, deep gray matter.

The MRE-parameters viscoelasticity | G^∗^|, elasticity *G*_*d*_, viscosity *G*_*l*_ and shear modulus phase angle Y were evaluated in the right and left hemispheres, cortices, corpus callosum and deep gray matter (Figures 3A,B). The comparison of the sonicated (right) with the contralateral (left) hemisphere revealed differences in viscoelasticity and elasticity 3 weeks after immunization in EAE group 3 (sonication 9 days after immunization with MOG, Supplementary Table 1). In these animals, mean values of viscoelasticity and elasticity were lower in the sonicated right hemisphere (mean | G^∗^| and *G*_*d*_ 6.1 and 4.9 kPa in the right vs. 7.2 and 5.7 kPa in the left hemisphere, adjusted *p*-value = 0.0026 for | G^∗^| and 0.0235 for *G*_*d*_; Figure 3C and Supplementary Table 1). The biomechanical properties of the sonicated right and normal left cortex were also different: animals in EAE group 3 (sonicated 9 days after EAE induction) exhibited lower viscosity of the right cortex both 2 and 3 weeks after immunization (mean cortical *G*_*l*_ 2.7 kPa on the right vs. 3.5 kPa on the left 2 weeks after FUS-BBBD and 2.1 vs. 2.9 kPa 3 weeks after FUS-BBBD, respectively; adjusted *p*-values = 0.0072 each; Figure 3C and Supplementary Table 1). Similarly, viscosity of the sonicated cortex was lower in group 2 EAE-animals that were sonicated 7 days after EAE-induction (mean *G*_*l*_ 2.9 kPa in the right vs. 3.6 kPa in the left cortex, adjusted *p*-value = 0.0399; Supplementary Table 1). Moreover, animals sonicated 9 days after immunization (group 3) had lower mean viscoelasticity and elasticity in the right cortex at the last imaging time point (mean | G^∗^| and *G*_*d*_ 5.9 and 5 kPa in the right vs. 7.5 and 6.5 kPa in the left cortex, adjusted *p*-value = 0.0040 for | G^∗^| and 0.0081 for *G*_*d*_; Supplementary Table 1). The shear modulus phase angle Y did not show different values in the right sonicated compared to the left normal hemisphere or cortex. Neither of the evaluated MRE-parameters revealed changes in the right corpus callosum or deep gray matter when comparing to the contralateral side (Supplementary Table 1). FUS-BBBD itself did not seem to affect cerebral biomechanics, as the MRE-parameters of control animals were similar in the sonicated and the contralateral brain parenchyma (Figure 3C and Supplementary Table 1).

A separate analysis focused on the longitudinal evolution of the biomechanical properties by comparing data acquired 2 and 3 weeks after immunization with MOG in animals that underwent FUS-BBBD on days 6 and 9, respectively (groups 1 and 3). Animals in EAE group 1 (FUS-BBBD 6 days after immunization with MOG) presented with a significant decrease of viscoelasticity in the sonicated hemisphere as well as the sonicated cortex over time (mean hemispherical | G^∗^| 7.2 vs. 6.3 kPa and mean cortical | G^∗^| 7.3 vs. 5.8 kPa 14 and 21 days after immunization, respectively; adjusted *p*-values 0.0371 and 0.0327). Moreover, elasticity and viscosity of the right sonicated hemisphere also decreased in this group when comparing the measurements from days 14 and 21 after EAE induction (mean *G*_*d*_ 5.7 vs. 4.9 kPa and mean *G*_*l*_ 3.6 vs. 3.1 kPa; adjusted *p*-values 0.0198 and 0.0147). Viscosity of the sonicated cortex dropped as well (mean *G*_*l*_ 3.2 vs. 2.3 kPa; adjusted *p*-value = 0.0248), while Y remained stable in all structures investigated. In addition, changes of viscoelasticity and viscosity could be observed in the contralateral normal cortex of animals in EAE group 1 (mean | G^∗^| 7.9 vs. 6.5 kPa and mean *G*_*l*_ 3.8 and 2.9 kPa; adjusted *p*-values 0.0459 and 0.151).

Animals in EAE group 3 (FUS-BBBD 9 days after EAE induction) only showed decreasing viscosity and phase angle in the sonicated hemisphere (mean *G*_*l*_ 3.3 vs. 2.8 kPa and mean Y 0.3834 vs. 0.3262; adjusted *p*-values 0.0263 and 0.0068).

In both EAE groups, biomechanical properties of the corpus callosum and the deep gray matter remained stable over time.

The comparison of mean viscoelasticity, elasticity, viscosity, and shear modulus phase angle in hyperintense T2w-conspicuities and contralateral NABT revealed no significant differences. However, mean elasticity of T2w-conspicuities trended to be lower than in NABT (mean *G*_*d*_ 5.5 vs. 6.1 kPa, *p*-value = 0.0507).

### Clusters of Activated Microglia/Macrophages Were Present in the Sonicated Hemisphere of Animals With EAE

**FIGURE 4:**
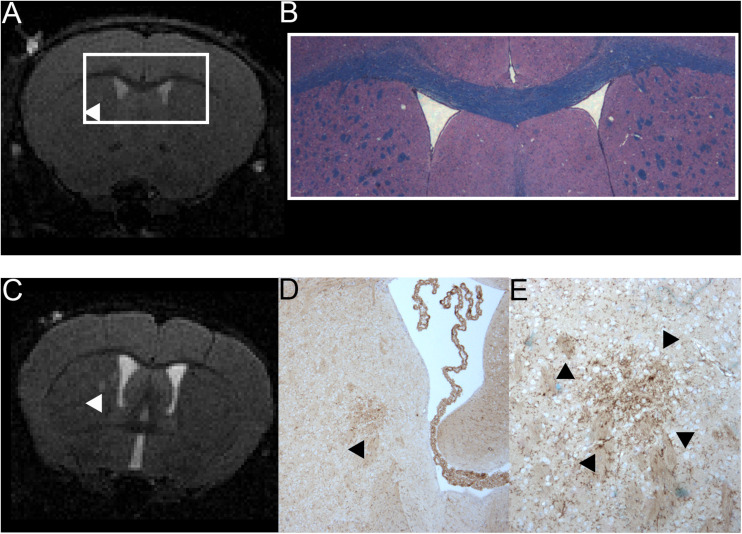
Comparison of MRI and H&E and LFB stain of one mouse with a T2w hyperintense lesion in the right thalamus and example of one animal with a T2w hyperintense lesion that could be co-localized to a cluster of resident microglia. A T2w image **(A)** and a H&E and LFB staining in 2-fold magnification **(B)** are shown. The white box in A frames the region depicted in panel **(B)**. The arrowhead in A indicates a T2w-conspicuity in the right thalamus. The H&E and LFB stain neither shows cell clustering nor obvious demyelination in this region. The images are from animal 9 in Tables 1, 2. A T2w image **(C)** and a TMEM119 staining in four-fold **(D)** and 10-fold magnification **(E)** are shown. The arrowheads indicate a T2w-conspicuity, which can be co-localized to a cluster of TMEM119-positive microglia [stained in brown; **(D,E)**]. The images are from animal 11 in Tables 1, 2.

**FIGURE 5:**
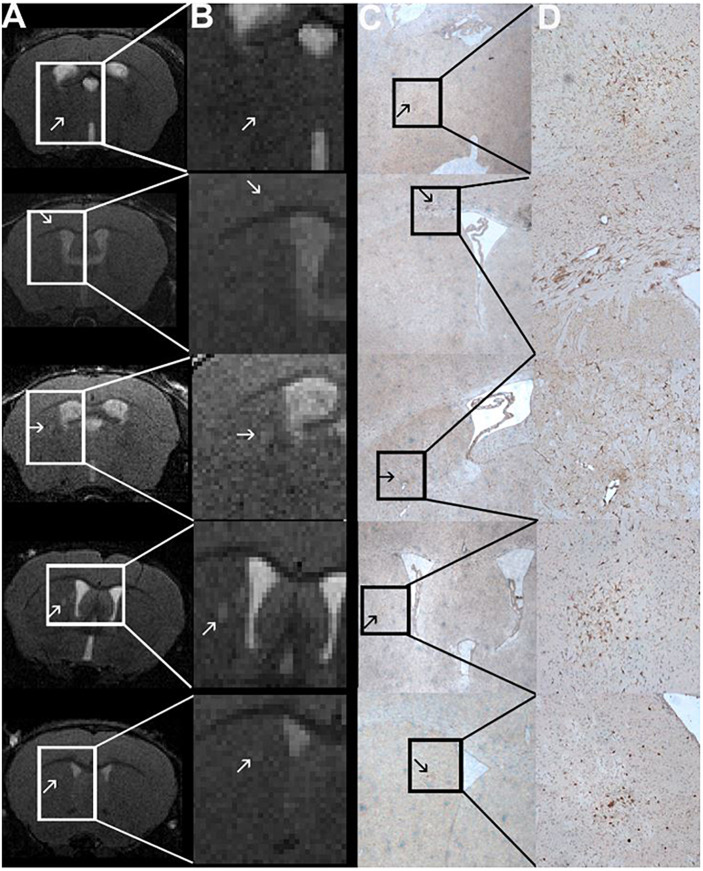
Comparison of MRI and Iba1-staining showing co-localized T2w lesions and clusters of activated macrophages/microglia. T2w images **(A)** with hyperintense lesions (indicated by the arrows). The abnormality-bearing region is framed by a box and magnified in panel **(B)**. Corresponding immune-histochemical Iba1-staining are depicted in C. Clusters of activated macrophages and microglia (stained in brown; arrows) can be co-localized to the T2w-lesions shown in panels **(A,B)**. The boxes in panel **(C)** indicate the area that is shown in 10-fold magnification in panel **(D)**. The images are from animals 1, 6, 9, and 11 in Tables 1, 2.

The sonicated brain parenchyma of all animals appeared normal on hematoxylin and eosin (H&E) stained slices (Table 2). Neither obvious tissue damage nor microbleeds could be observed. EAE animals did not show any larger cellular infiltrates in the sonicated area on H&E staining. Moreover, there was no obvious demyelination identifiable in this region when examining the luxol fast blue (LFB) staining (Figures 4A,B). Immune-histochemical staining identified foci of Iba1-positive microglia/macrophages in the sonicated right hemisphere of seven EAE animals (7/12, 58.3%; Table 2). Of note, all animals that were sonicated 9 days after immunization presented with such accumulations of activated microglia/macrophages (4/4, 100%). Again, half of the animals (2/4, 50%) sonicated 7 days after EAE induction had foci of Iba-1 positive microglia/macrophages, while only one animal (1/4, 25 %) showed this alteration when FUS was performed 6 days after immunization. These foci could be co-localized by their morphology to five out of eight T2w-conspicuities (Figure 5). Control animals did not present with accumulation of Iba1-positive microglia/macrophages (Table 2).

**TABLE 2:** Summary of histopathological findings in the sonicated area of animals with EAE and healthy controls.

Animal	FUS performed after immunization (days)	H&E		LFB	Iba-1	TMEM119
		Hemorrhage	Cell infiltration	Demye lination	Foci of activated microglia/macrophages	Microglial marker
**EAE**						
1*	6	0	0	0	1	0
2	6	0	0	0	0	0
3	6	0	0	0	0	0
4	6	0	0	0	0	0
5	7	0	0	0	0	0
6*	7	0	0	0	1	0
7	7	0	0	0	1	0
8	7	0	0	0	0	0
9*	9	0	0	0	1	0
10	9	0	0	0	1	1
11*^#^	9	0	0	0	1	1
12	9	0	0	0	1	1
**Control**						
1	n/a	0	0	0	0	0
2	n/a	0	0	0	0	0
3	n/a	0	0	0	0	0
4	n/a	0	0	0	0	0

*The asterisks indicate the animals with clusters of activated macrophages and microglia that could be co-localized to T2w-lesions. The hashtag indicates the animal with a cluster of Iba1- and TMEM119-positive activated resident microglia that could be co-localized to a hyperintense T2w-lesion. FUS, focused ultrasound.*

The additional TMEM119-staining revealed that three of the five clusters of Iba1-positive cells also show increased expression of TMEM119, which indicated that they consist, at least in part, of resident microglia and not only of infiltrating macrophages (Table 2). The foci of Iba1- and TMEM119-positive resident microglia could exclusively be observed in animals sonicated 9 days after immunization. Neither animals sonicated 6 or 7 days after EAE inductions nor controls showed these reactive TMEM119-positive cells. In these animals microglia remained in their resting state. In one animal, the cluster of reactive resident microglia could be co-localized to a T2w-conspicuity (Figures 4C–E).

## Discussion

In this study, we observed focal T2w-hyperintense abnormalities corresponding to microglia/macrophage accumulation in the sonicated brain tissue of mice immunized with MOG. This was accompanied by differences in MRE parameters when comparing the right sonicated hemisphere and cortex to the contralateral normal side. Focal T2w signal alterations, changes in biomechanical properties and microglia/macrophage activation were most common in those animals in which BBBD was induced 9 days after immunization.

T2-weighted-hyper- and T1w-isointense abnormalities could be detected in the sonicated hemisphere of seven EAE animals. Nessler et al. (2007) analyzed MRI characteristics and their histopathological correlates in adoptive transfer EAE in SJL/J mice. They observed two lesion types: type A lesions were T1w- and T2w-hypointense and showed a high density of inflammatory cells and loss of myelin as well as axons. Type B lesions were T2w-hyperintense and T1w-iso- or mildly hypointense, and were characterized by a moderate inflammatory cell infiltration, a moderate amount of myelin and axonal loss and prominent immunoglobulin (Ig) deposition (Nessler et al., 2007). Moreover, T2w hyperintensity of lesions was positively correlated with the density of activated microglia and reactive astrocytes. Microglia activation did not correlate with contrast-enhancement. Our MRI and histological findings in FUS-induced EAE lesions are consistent with Type B lesions according to Nessler et al. (2007).

Magnetic resonance elastography reveals altered biomechanical properties of the brain in animal models of MS (Riek et al., 2012; Schregel et al., 2012; Millward et al., 2015; Wang et al., 2020) and in human patients (Wuerfel et al., 2010; Streitberger et al., 2012; Fehlner et al., 2015). In EAE, changes in viscoelasticity correlated with the magnitude of inflammation (Riek et al., 2012; Millward et al., 2015). In this study, we observed lower viscoelasticity, elasticity and viscosity in the sonicated compared to the contralateral hemispheres and cortices of animals with EAE. Control animals that underwent FUS-BBBD but were not immunized with MOG did not show changes of MRE parameters. Thus, MRE seems to capture changes in brain biomechanics caused by EAE in combination with FUS-BBBD. Millward et al. (2015) observed a correlation between F4/80 gene expression as marker of macrophages/microglia and elasticity reduction. Presence of activated macrophages/microglia in our study affected elasticity in a similar way. Moreover, we found that viscoelasticity and viscosity were affected by early inflammatory changes as well.

Microglia are crucial for the development of EAE, as pharmacologically induced microglial paralysis represses symptoms and CNS inflammation (Heppner et al., 2005). Cytokines and chemokines released by activated microglia attract and activate other immune cells and reactive oxygen species can contribute to tissue damage (Heppner et al., 2005). Alvarez et al. investigated early BBB changes and immune cell activation in a mouse model of spontaneous relapsing-remitting EAE prior to the onset of symptoms (Alvarez et al., 2015). The authors demonstrated impairment of the BBB well before T-cell infiltration and demyelination occurred and described perivascular accumulation of macrophages and microglia from an early time point on (Alvarez et al., 2015). The presence of macrophages and microglia increased with time, while modest T-cell and leukocyte infiltration was only observed at later pre-symptomatic stages (Alvarez et al., 2015). Maggi et al. (2014) observed focal increased BBB permeability in areas of future lesion development in a marmoset EAE model. These permeability changes corresponded to small non-demyelinated inflammatory nodules consisting of activated microglia and a variable number of lymphocytes, which were barely or not visible on T2w MRI (Maggi et al., 2014). Based on their results, the authors hypothesized that such inflammatory foci precede demyelinated lesions.

Bolton and Smith (2015) reviewed the acute inflammatory events during lesion formation in EAE and state that microglia are critical for both instigation and control of the disease. In this context, our model is exquisitely suited to further explore the temporal sequence and the mediators involved in lesion formation.

Clusters of activated microglia have been found in the brain of patients with MS (De Groot et al., 2001) as well, irrespective of disease duration, type, and gender (van Horssen et al., 2012). These nodules were accompanied by a variable degree of edema, did neither show apparent demyelination nor infiltration of other immune cells and were detectable with conventional MRI (De Groot et al., 2001). Such foci have been termed (p)reactive lesions. It has been discussed that they reflect an early disease stage preceding the occurrence of a demyelinating MS lesion (van der Valk and Amor, 2009), even though some (p)reactive lesions are likely to resolve spontaneously (van Horssen et al., 2012). We have also described T2w hyperintensity preceding gadolinium enhancement on T1w images in a subset of newly occurring lesions in human MS patients followed by weekly MRI exams, supporting the temporal sequence inferred by previous work (Guttmann et al., 2016). Nodules of activated microglia have also been shown to be associated with damaged axons (Singh et al., 2013) and do not seem to be specific for MS.

Neither MRI nor histopathology showed gross tissue damage, macro- or microhemorrhages after FUS-BBBD. However, a recent study by Kovacs et al. demonstrated that BBBD by FUS induces a transient sterile inflammatory response (SIR) similar to that occurring with ischemia or trauma (Kovacs et al., 2017). The authors observed a rapidly induced damage-associated molecular pattern including proinflammatory cytokines and transcriptomic changes of the NFκB pathway, which lasted for 12–24 h after sonication. Additionally, increased numbers of apoptotic TUNEL^+^ cells and activated astrocytes could be found up to 24 h after FUS-BBBD (Kovacs et al., 2017). Moreover, systemic macrophages were seen in sonicated parenchyma after 6 days. Even though our predominant histopathological finding similarly consisted of focal accumulation of activated macrophages and microglia, it is unlikely that this was only due to an SIR following FUS-BBBD. First, the changes we observe follow a different time course than that described in SIR. The microglia activation following sonication observed by Kovacs et al. (2017) resolved after 24 h, while it was seen on histopathological analysis performed 2 weeks after FUS-BBBD in our study. Second, the control animals in our study did not present with accumulation of Iba1-positive microglia/macrophages, while SIR following sonication was reported in healthy animals (Kovacs et al., 2017). Third, Kovacs et al. (2017) used an atypically high dose of microbubbles (100 μl). The effects described have not been observed with the microbubble concentration we used (100 μl/kg) when applying comparable acoustic parameters. Hence, the activation of microglia and macrophages observed in our experiments presumably occurred in the context of EAE in combination with FUS-BBBD and was not caused by FUS-induced SIR or axonal damage.

In pre-symptomatic mice with spontaneous relapsing-remitting EAE a peripheral pro-inflammatory response of immune cells was observed before BBBD or any other disease-related CNS pathology (Alvarez et al., 2015). This suggests an influence of peripheral immune activation on inflammatory responses within the CNS (Alvarez et al., 2015) and might explain why the T2w signal abnormalities and clusters of activated microglia/macrophages were most commonly seen in those animals in which BBBD was induced 9 days after immunization with MOG. In this subgroup of animals, peripheral immune responses were presumably more advanced than in the other animals, increasing the likelihood of a promoted CNS inflammation.

In three animals sonicated 9 days after EAE induction clusters of Iba1- and TMEM119-positive cells could be identified, which indicates that these clusters include resident microglia (Bennett et al., 2016; Satoh et al., 2016). Resident microglia and infiltrating macrophages respond differently to acute inflammatory stimuli and have been described to exert differential functions in EAE (Yamasaki et al., 2014). It has been suggested that diffusible stimuli such as complement and antibodies are involved in microglial activation in EAE (Caravagna et al., 2018). Moreover, leakage of fibrinogen through an impaired BBB triggered perivascular microglial clustering in the spinal cord of mice with EAE before symptom onset (Davalos et al., 2012). Thus, FUS-BBBD could potentially enhance the presence of such stimuli in the brain of EAE-animals and thereby promote the activation of microglia.

The clinical phenotype of the EAE animals was mild in general, and the subgroup of animals that underwent MRI under isoflurane anesthesia at the earlier time point (9 days after immunization) showed a milder clinical phenotype than the rest of the EAE animals. It has been shown previously that the clinical severity of EAE is attenuated by sevoflurane (Polak et al., 2012), as inhaled anesthetics can suppress T-cell activation. This might explain why these animals only developed very mild symptoms. To avoid this issue in the other two groups, we did not perform MRI at 9 days post-immunization and isoflurane anesthesia was used only after first occurrence of symptoms. Overall, the clinical time course with observation of first symptoms 11 days after induction falls into the range previously described for MOG-EAE (Brown and Sawchenko, 2007; Bittner et al., 2014). Symptoms progressed to a maximum paraplegia of the hind limbs. Even though more severe symptoms with animals reaching a moribund condition can occur, the disease course we observed is similar to published kinetics of MOG-induced EAE (Bittner et al., 2014; Barthelmes et al., 2015). However, we cannot exclude that the required anesthesia alone or in combination with FUS-BBBD had an influence on the clinical phenotype and/or the effector cells in our experiment.

Limitations of our study include the small sample size, the use of only one EAE model, as well as the lack of more extensive histopathological characterization. Future studies using different mouse strains and myelin peptides as well as adoptive transfer EAE are warranted to confirm and extend our findings. Additionally, the effect of repeated sonication of immunized animals, and hence repeated BBBD, on lesion occurrence and evolution should be studied. Furthermore, the immune response to FUS-BBBD in different EAE-models should be evaluated in more detail. Moreover, general anesthesia is required for FUS and imaging. The potential impact of anesthetic regimens on effector cells, lesion development and disease course should be investigated in detail. Future work should also include analyses of Ig deposition in order to further clarify the mechanism of microglial activation. Safety, kinetics and tissue integrity in the context of FUS-BBBD have extensively been studied in various animal models over the past few years (Hynynen et al., 2005; Baseri et al., 2010; Howles et al., 2010; Konofagou, 2012; Marty et al., 2012; Aryal et al., 2014; Alli et al., 2018). Our results suggest that comparable safety profiles and BBBD-kinetics apply for animals with EAE. However, to our knowledge this is the first study applying FUS in EAE and more extensive analyses of the direct effects of FUS on brain tissue and blood barrier in the setting of EAE should be performed. Our FUS-driven model harbors the advantage that surgery, a necessity for other methods (Westland et al., 1999; Woodruff and Franklin, 1999; Newman et al., 2001; Kerschensteiner et al., 2004), is not required for targeted lesion induction. Overall, the work we present here is a proof-of-concept study. Future experiments including analyses of the effects of FUS on the BBB and the clinical course in different EAE-models along with an extensive histopathological and molecular characterization are needed to lend our approach robustness and to establish it as a reproducible model of focal lesion development in EAE.

In conclusion, we here provide first evidence for a controlled lesion induction model in EAE using FUS-BBBD. Focal activation of microglia/macrophages within the sonicated parenchyma of MOG immunized animals may be interpreted as targeted initial inflammatory activity, which can be identified and monitored with MRI and MRE.

## Data Availability Statement

The raw data supporting the conclusions of this article will be made available by the authors, without undue reservation.

## Ethics Statement

The animal study was reviewed and approved by the Institutional Animal Care and Use Committee (IACUC) Brigham and Women’s Hospital Boston, MA, United States.

## Author Contributions

CG and KS conceptualized the experiments. CB, RM, KS, and MP were involved in EAE induction and clinical monitoring. KS and MP acquired MRI and MRE data. PW, YZ-Z, and NM performed focused-ultrasound. CB and YZ-Z performed histopathology. RS supported MRE data acquisition, reconstruction, and interpretation. KS, PW, CB, JW, PF, RS, and CG analyzed and interpreted the data. KS, CG, CB, JW, and PW wrote the manuscript. All authors contributed to the article and approved the submitted version.

## Conflict of Interest

CG has received research funding from Sanofi, the National Multiple Sclerosis Society, and the International Progressive Multiple Sclerosis Alliance, the U.S. Office for Naval Research, as well as travel support from Roche Pharmaceuticals; CG owns stock in Roche, Novartis, GSK, Alnylam, Protalix Biotherapeutics, Arrowhead Pharmaceuticals, Cocrystal Pharma, Sangamo Therapeutics, Alcon. JW is the CEO of MIAC AG Basel, Switzerland. He served on scientific advisory boards of Actelion, Biogen, Genzyme-Sanofi, Novartis, and Roche. He is or was supported by grants of the EU (Horizon2020), German Federal Ministries of Education and Research (BMBF) and of Economic Affairs and Energy (BMWI). The remaining authors declare that the research was conducted in the absence of any commercial or financial relationships that could be construed as a potential conflict of interest.
