# Supplementary material for: Targeted Blood Brain Barrier Opening With Focused Ultrasound Induces Focal Macrophage/Microglial Activation in Experimental Autoimmune Encephalomyelitis
Source: Front Neurosci. 2021 May 12;15:665722. doi: 10.3389/fnins.2021.665722 (PMC8149750; doi:10.3389/fnins.2021.665722)
Supplement: Supplementary file 1 [file Table_1.docx]

| **group** | **right sonicated hemisphere** | **left normal hemisphere** | **adjusted p-value** | **right sonicated cortex** | **left normal cortex** | **adjusted p-value** | **right sonicated corpus callosum** | **left normal corpus callosum** | **adjusted p-value** | **right sonicated deep grey matter** | **left normal deep grey matter** | **adjusted p-value** |
| --- | --- | --- | --- | --- | --- | --- | --- | --- | --- | --- | --- | --- |
|  | **viscoelasticity lG*l in kPa [mean (SD)]** | | | | | | | | | | | |
| EAE group 1 MRE d14 | 7.19  (0.22) | 6.99  (0.30) | > 0.9 | 7.25  (0.71) | 7.88  (0.21) | > 0.9 | 6.89  (0.67) | 6.34  (0.40) | > 0.9 | 9.12  (0.67) | 8.07  (0.56) | 0.2641 |
| EAE group 1 MRE d21 | 6.26  (0.30) | 6.32  (0.42) | > 0.9 | 5.83  (0.15) | 6.54  (0.62) | 0.9844 | 6.80  (0.13) | 6.97  (0.55) | > 0.9 | 7.97  (1.32) | 6.65  (0.76) | 0.0815 |
| EAE group 2 MRE d9 | 7.96  (0.73) | 7.82  (0.42) | > 0.9 | 7.28  (1.43) | 8.14  (0.48) | 0.5455 | 6.21  (1.28) | 5.92  (0.61) | > 0.9 | 7.75  (1.52) | 7.06  (0.70) | > 0.9 |
| EAE group 2 MRE d14 | 7.05  (0.34) | 7.16  (0.09) | > 0.9 | 6.72  (1.08) | 7.73  (1.15) | 0.3028 | 6.79  (0.43) | 6.56  (0.22) | > 0.9 | 6.36  (0.71) | 6.28  (0.31) | > 0.9 |
| EAE group 3 MRE d14 | 6.40  (0.30) | 6.70  (0.25) | > 0.9 | 6.33  (0.89) | 7.12  (0.82) | 0.4487 | 6.96  (0.35) | 6.84  (0.13) | > 0.9 | 6.72  (0.65) | 6.07  (0.40) | 0.8746 |
| EAE group 3 MRE d21 | 6.11  (0.55) | 7.18  (0.56) | 0.0026 | 5.82  (0.44) | 7.52  (0.56) | 0.0040 | 6.99  (0.78) | 7.64  (0.68) | 0.4530 | 6.60  (0.47) | 6.04  (1.24) | > 0.9 |
| controls | 7.09  (0.46) | 7.09  (0.12) | > 0.9 | 6.77  (0.48) | 7.47  (0.14) | > 0.9 | 7.43  (0.74) | 7.12  (0.45) | > 0.9 | 7.70  (0.56) | 6.73  (0.60) | 0.3687 |
|  | **elasticity G_d_ in kPa [mean (SD)]** | | | | | | | | | | | |
| EAE group 1 MRE d14 | 5.69  (0.17) | 5.63  (0.35) | > 0.9 | 6.06  (0.54) | 6.43  (0.42) | > 0.9 | 5.39  (0.69) | 5.14  (0.37) | > 0.9 | 6.56  (0.50) | 6.11  (0.16) | > 0.9 |
| EAE group 1 MRE d21 | 4.93  (0.26) | 5.01  (0.53) | > 0.9 | 4.97  (0.29) | 5.39  (0.84) | > 0.9 | 5.36  (0.46) | 5.69  (0.67) | > 0.9 | 6.09  (0.94) | 5.21  (0.84) | 0.4854 |
| EAE group 2 MRE d9 | 6.03  (0.63) | 6.07  (0.27) | > 0.9 | 5.90  (1.00) | 6.58  (0.67) | 0.9213 | 5.10  (1.22) | 4.78  (0.44) | > 0.9 | 5.94  (1.07) | 5.67  (0.59) | > 0.9 |
| EAE group 2 MRE d14 | 5.50  (0.29) | 5.72  (0.11) | > 0.9 | 5.64  (0.94) | 6.39  (1.06) | 0.6906 | 5.63  (0.55) | 5.30  (0.20) | > 0.9 | 4.77  (0.40) | 5.02  (0.43) | > 0.9 |
| EAE group 3 MRE d14 | 4.91  (0.18) | 5.30  (0.21) | 0.8040 | 5.21  (0.78) | 5.81  (0.66) | 0.8629 | 5.69  (0.39) | 5.63  (0.27) | > 0.9 | 4.95  (0.56) | 4.73  (0.35) | > 0.9 |
| EAE group 3 MRE d21 | 4.88  (0.41) | 5.70  (0.48) | 0.0235 | 5.02  (0.55) | 6.47  (0.39) | 0.0081 | 5.84  (0.69) | 6.40  (0.58) | 0.7208 | 5.09  (0.36) | 4.70  (1.30) | > 0.9 |
| controls | 5.53  (0.44) | 5.58  (0.29) | > 0.9 | 5.23  (0.45) | 6.07  (0.57) | 0.4693 | 6.13  (0.61) | 5.74  (0.50) | > 0.9 | 6.17  (0.75) | 5.13  (0.46) | 0.2393 |
|  | **viscosity G_l_ in kPa [mean (SD)]** | | | | | | | | | | | |
| EAE group 1 MRE d14 | 3.63  (0.27) | 3.55  (0.39) | > 0.9 | 3.21  (0.37) | 3.83  (0.43) | 0.0995 | 3.66  (0.64) | 3.17  (0.32) | 0.6425 | 5.33  (0.25) | 4.36  (1.05) | 0.1351 |
| EAE group 1 MRE d21 | 3.07  (0.09) | 3.25  (0.04) | > 0.9 | 2.32  (00.28) | 2.87  (0.16) | 0.1968 | 3.76  (0.51) | 3.46  (0.25) | > 0.9 | 4.44  (0.90) | 3.42  (0.21) | 0.1010 |
| EAE group 2 MRE d9 | 4.26  (0.38) | 4.87  (1.04) | 0.4026 | 3.47  (0.80) | 3.80  (0.90) | > 0.9 | 3.16  (0.52) | 3.00  (0.41) | > 0.9 | 4.15  (0.83) | 3.67  (0.53) | > 0.9 |
| EAE group 2 MRE d14 | 3.61  (0.35) | 3.62  (0.09) | > 0.9 | 2.86  (0.33) | 3.59  (0.24) | 0.0399 | 3.19  (0.17) | 3.31  (0.32) | > 0.9 | 3.52  (0.66) | 3.24  (0.08) | > 0.9 |
| EAE group 3 MRE d14 | 3.28  (0.17) | 3.47  (0.15) | > 0.9 | 2.66  (0.35) | 3.45  (0.60) | 0.0072 | 3.45  (0.45) | 3.26  (0.32) | > 0.9 | 3.80  (0.27) | 3.28  (0.43) | 0.8969 |
| EAE group 3 MRE d21 | 2.84  (0.19) | 3.55  (0.20) | 0.0957 | 2.13  (0.16) | 2.92  (0.31) | 0.0072 | 3.32  (0.47) | 3.53  (0.64) | > 0.9 | 3.59  (0.50) | 3.42  (0.36) | > 0.9 |
| controls | 3.38  (0.22) | 3.63  (0.15) | > 0.9 | 3.09  (0.47) | 3.38  (0.35) | > 0.9 | 3.64  (0.51) | 3.32  (0.44) | > 0.9 | 3.78  (0.33) | 3.87  (0.35) | > 0.9 |
|  | **phase angle Y [mean (SD)]** | | | | | | | | | | | |
| EAE group 1 MRE d14 | 0.366  (0.02) | 0.362  (0.05) | > 0.9 | 0.329  (0.03) | 0.369  (0.06) | 0.6455 | 0.374  (0.06) | 0.339  (0.03) | > 0.9 | 0.443  (0.01) | 0.406  (0.05) | > 0.9 |
| EAE group 1 MRE d21 | 0.365  (0.04) | 0.381  (0.04) | > 0.9 | 0.314  (0.08) | 0.336  (0.05) | > 0.9 | 0.386  (0.07) | 0.343  (0.04) | 0.5316 | 0.413  (0.02) | 0.380  (0.04) | > 0.9 |
| EAE group 2 MRE d9 | 0.396  (0.004) | 0.379  (0.04) | > 0.9 | 0.355  (0.03) | 0.354  (0.07) | > 0.9 | 0.348  (0.02) | 0.346  (0.02) | > 0.9 | 0.391  (0.03) | 0.352  (0.04) | > 0.9 |
| EAE group 2 MRE d14 | 0.379  (0.02) | 0.364  (0.01) | > 0.9 | 0.318  (0.02) | 0.335  (0.05) | > 0.9 | 0.333  (0.03) | 0.350  (0.02) | > 0.9 | 0.404  (0.03) | 0.379  (0.02) | > 0.9 |
| EAE group 3 MRE d14 | 0.383  (0.01) | 0.379  (0.02) | > 0.9 | 0.334  (0.04) | 0.365  (0.05) | 0.9000 | 0.351  (0.04) | 0.334  (0.03) | > 0.9 | 0.389  (0.05) | 0.374  (0.03) | > 0.9 |
| EAE group 3 MRE d21 | 0.326  (0.02) | 0.356  (0.03) | 0.4501 | 0.269  (0.05) | 0.274  (0.03) | > 0.9 | 0.341  (0.02) | 0.318  (0.04) | > 0.9 | 0.382  (0.05) | 0.394  (0.03) | > 0.9 |
| controls | 0.382  (0.01) | 0.379  (0.03) | > 0.9 | 0.378  (0.05) | 0.356  (0.05) | > 0.9 | 0.336  (0.02) | 0.335  (0.04) | > 0.9 | 0.372  (0.04) | 0.389  (0.01) | > 0.9 |

**Supplementary table 1: Summary of MRE findings in animals with EAE and healthy controls.** FUS-BBBD was performed 6, 7 and 9 days after EAE induction in subgroups (EAE group 1,2 and 3) as well as in healthy controls. In EAE groups 1 and 3, MRI and MRE data were acquired 14 and 21 days after immunization and in EAE group 2 after 9 days to capture potential early changes and after 14 days. The biomechanical properties viscoelasticity lG*l, elasticity G_d_, viscosity G_l_ and phase angle Y were assessed in the hemispheres, cortices, corpora callosum and deep grey matter of animals immunized with MOG and in healthy controls. Mean and standard deviation of each parameter are reported. Two-way ANOVA followed by Bonferroni’s test for multiple comparisons was performed to compare the right sonicated side to the contralateral normal side. Adjusted p-values from Bonferroni’s test are shown. d, day; FUS, focused ultrasound; FUS-BBBD, FUS-induced blood brain barrier breakdown; SD, standard deviation.
